# Comparative growth dynamics of bacterial and fungal contaminants in bupivacaine liposomal injectable suspension, bupivacaine 0.5%, and propofol

**DOI:** 10.1371/journal.pone.0281768

**Published:** 2023-02-16

**Authors:** Amber Wallace, Lydia Love, Catherine Gensler, Megan Jacob, James Robertson, Kristen Messenger

**Affiliations:** 1 Department of Molecular Biomedical Sciences, North Carolina State University College of Veterinary Medicine, Raleigh, North Carolina, United States of America; 2 Department of Population Health and Pathobiology, North Carolina State University College of Veterinary Medicine, Raleigh, North Carolina, United States of America; 3 Biostatistics Administrative Department, North Carolina State University College of Veterinary Medicine, Raleigh, North Carolina, United States of America; Universidad Autonoma de Chihuahua, MEXICO

## Abstract

**Objective:**

To determine whether bupivacaine liposomal injectable suspension (BLIS) supports microbial growth when artificially inoculated and to evaluate liposomal stability in the face of this extrinsic contamination as evidenced by changes in free bupivacaine concentrations.

**Study design:**

A randomized, prospective *in vitro* study in which three vials of each BLIS, bupivacaine 0.5%, and propofol were individually inoculated with known concentrations of *Escherichia coli*, *Pseudomonas aeruginosa*, *Staphylococcus aureus*, and *Candida albicans* (n = 36) to quantify bacterial and fungal growth was conducted. Over 120 hours, aliquots from contaminated vials were withdrawn, plated, and incubated to determine microbial concentrations. High-pressure liquid chromatography (HPLC) was used to evaluate free bupivacaine concentrations over time in BLIS. Data were analyzed using a mixed effects model with multiple comparisons.

**Sample population:**

Twelve vials of each BLIS, bupivacaine 0.5%, and propofol.

**Results:**

BLIS did not support significant growth of *Staphylococcus aureus* or *Candida albicans* at any time. BLIS supported significant growth of *Escherichia coli* and *Pseudomonas aeruginosa* beginning at the 24 hour time point. Bupivacaine 0.5% did not support significant growth of any organisms. Propofol supported significant growth of all organisms. Free bupivacaine concentrations changed minimally over time.

**Conclusion:**

Bacterial and fungal contaminant growth in artificially inoculated BLIS is organism dependent. BLIS supports significant growth of *Escherichia coli* and *Pseudomonas aeruginosa*. Extra-label handling of BLIS should only be undertaken with caution and with adherence to strict aseptic technique.

## Introduction

Adequate pain management for veterinary species is essential in the provision of humane perioperative care. Injectable opioid analgesics are often prescribed for acute surgical pain, although they may produce respiratory, cardiovascular, and gastrointestinal side effects [1]. Oral formulations of opioids offer limited bioavailability, minimizing their usefulness on an outpatient basis [2–4]. Additionally, the ongoing human opioid crisis, wherein the abuse of opioids can lead to fatal overdoses [5], may make outpatient opioid use in veterinary species a less attractive option for pain management. Non-steroidal anti-inflammatories are another powerful option available for pain management but unfortunately have numerous relative contraindications [6], are variably effective [7], and may be toxic to some domestic species [8]. The safe and economical incorporation of local anesthetics into multimodal pain management may reduce the opportunity for opioid diversion as well as dependence on other types of analgesics, thereby minimizing the risk of adverse drug effects and improving the quality of patient care. Unfortunately, many local anesthetics have a limited duration of action and do not provide prolonged analgesia.

Bupivacaine liposome injectable suspension (BLIS; Nocita, Elanco, Greenfield, IN) is a preservative-free, local anesthetic solution of multi-vesicular lipid-based particles containing bupivacaine that is labeled for post-operative analgesia for canine cranial cruciate ligament surgery and feline onychectomy procedures. Lipid encapsulation of bupivacaine allows the gradual release of local anesthetic, prolonging the blockade of sodium channels and providing local analgesia for up to 72 hours. The Food and Drug Administration approved label states that a vial of BLIS should be punctured once, and multiple, sterile syringes can be filled and stored at room temperature for a maximum of four hours [9]. These storage and handling specifications may result in significant waste and limit the economical use of BLIS, particularly in clinical settings with a small daily caseload. In the United States, extra-label drug use is legal and common practice, and extra-label use of BLIS with multiple vial punctures over consecutive days may be a cost effective alternative for using this product. However, in the authors’ experience, the extra-label use of BLIS varies between veterinary practitioners and institutions. In addition, there is minimal data on the growth of common pathogens in BLIS over prolonged periods of time or the effect of contamination on the integrity of the multivesicular liposomes. Carlson et. al demonstrated that multiple vial punctures over consecutive days did not result in microbial contamination, and the concentration of unencapsulated bupivacaine did not vary significantly for four days [10].

Here, we investigated the growth dynamics of *Escherichia coli (E*. *coli)*, *Pseudomonas aeruginosa (P*. *aeruginosa)*, *Staphylococcus aureus (S*. *aureus)*, *and Candida albicans (C*. *albicans)* in artificially inoculated BLIS in comparison to propofol and 0.5% bupivacaine. The concentration of unencapsulated bupivacaine in BLIS was also quantified following this extrinsic contamination. We hypothesized that, when extrinsically contaminated, BLIS would be both bacterio- and fungistatic and that unencapsulated concentrations of bupivacaine would remain unchanged.

## Materials and methods

### Part one: Comparative microbial growth dynamics

To confirm the absence of contamination at the start of the study, twelve 20 mL vials of each BLIS, propofol, and 0.5% bupivacaine (n = 36) were punctured aseptically with 18-gauge needles. Each time a vial was punctured, the rubber septum of each vial was disinfected with alcohol, and a new sterile needle and syringe were used. Aliquots (250 μL) were removed from each vial, and 10 μL of undiluted samples were plated in triplicate onto trypticase soy agar with 5% Columbia sheep blood agar plates (BAP; Remel, Lenexa, KS) following a four-quadrant drop-plate technique (a cost effective strategy wherein multiple locations on a singular BAP can be used to evaluate organism growth) [11]. The BAPs were incubated at 37°C for 24 hours and evaluated for growth of any organism via manual inspection and quantification of colony forming units (CFUs).

The *in vitro* growth of four different organisms originating from the American Type Culture Collection (ATCC, Manassas, KA) were investigated; specifically, *E*. *coli* (ATCC 25922), *P*. *aeruginosa* (ATCC 27853), *S*. *aureus* (ATCC 25923), and *C*. *albicans* (ATCC 14053) were used. Cultures were established in October 2022 from freezer stocks of each organism onto BAP and incubated for 24 hours at 37°C for bacteria and 48 hours at 25°C for yeast.

To prepare inoculates, a single colony of each organism was added to sterile phosphate buffered saline (PBS) and adjusted to a standardized turbidity of 0.5 McFarland units (Vitek Densichek, Biomireux, France). To determine the specific starting concentration of each organism, an aliquot of each suspension was serially diluted tenfold in PBS, and each dilution was spot plated (10μL) in triplicate. BAPs were incubated as described above, and the colonies were counted and log-transformed to determine the starting CFU/ml.

Three vials of each drug (n = 9) were then artificially inoculated with a 1:100 dilution of the starting inoculum such that each vial of drug was inoculated with an individual microorganism at a calculated target concentration of 2x10^4^ CFU/mL. This starting concentration is similar to that utilized by Sosis and Braverman [12]. Vials were vortexed following inoculation and stored at room temperature (~25°C) for the duration of the study. At each designated time point, 0 hours (immediately following inoculation), 3 hours, 6 hours, 12 hours, 24 hours, and then every 24 hours thereafter for 120 hours post-inoculation, contaminated vials were vortexed and 250 μL aliquots were removed using aseptic technique. Aliquots were serially diluted tenfold in PBS, plated in triplicate, and incubated as previously described. Following 24 hours of incubation, a manual count of colony forming units was collected. The triplicate count of the highest dilution with a countable range (target 20 to 200 CFU) was averaged for each drug prior to statistical analysis.

### Part two: Unencapsulated bupivacaine concentrations

During the microbiologic portion of the study, aliquots (150 μL) of BLIS were collected from each vial (n = 12), beginning prior to inoculation and every 24 hours thereafter for 120 hours, and analyzed by high-pressure liquid chromatography to evaluate changes in the concentration of unencapsulated bupivacaine. Aliquots were placed into 1 mL polypropylene vials and immediately centrifuged at 3500g for 10 minutes at 25°C to separate liposomes from free bupivacaine [10]. One hundred microliters of supernatant were collected from each centrifuged vial and stored at -80°C until analysis by HPLC.

All reagents were of analytical, HPLC, or liquid chromatography-mass spectrometry grade. Acetonitrile was supplied by Fisher Chemical (Raleigh, NC). Trifluoroacetic acid and bupivacaine hydrochloride reference standards were purchased from Sigma Aldrich (St. Louis, MO). Ultrapure water was supplied by Waters Corporation (Milford, MA).

Bupivacaine concentrations were analyzed using HPLC with ultraviolet detection. The HPLC system consisted of a Waters 2965 separation module and Waters 996 detector (Waters Corporation, Milford, MA), set to a wavelength of 210 nm. Bupivacaine was separated by a C18 XBridge 3.5 μm (4.6 x100 mm) column maintained at 35°C. The mobile phase consisted of phase A (0.02% trifluoroacetic acid in water) and 100% acetonitrile of phase B (60:40). The flow rate was set to 0.8 mL/min with a run time of five minutes. Injection volume was 50 μl with two minutes of retention time. Quality controls (1.5, 3 and 7 μg/mL) and standards (0.05–10 μg/mL) were made in ultrapure-water. Intra-day precision was 0.39–4.1% with 87.9–99.2% of recovery. The limit of quantification was recognized as 0.5 μg/mL. The limit of detection was 0.05 μg/mL. Samples were diluted in ultrapure-water at 100–200 folds and filtered using 0.2μm, 13 mm polyvinylidine fluoride syringe filters (Pall Corporation, Westbourough, MA) before analysis.

### Statistical analysis

To determine whether each drug affected the rates of microbial growth in the drug vials, a linear mixed model was fit with fixed effects of time, time squared, and the drug.

A random intercept was included for each bottle within each set of three plates. The response was the log-transformed cell counts where all cell counts had one added to allow for zero values. Backward selection was done via the Akaike information criteria to examine the utility of removing the higher order time term and the interactions.

For comparisons of drugs within time points, the Welch-corrected *t* test was used with Bonferroni correction applied within each time point for the three tests applied. For the detection of changes from baseline, a progressive Bonferroni correction was applied such that starting from the earliest time point each comparison was corrected for the number of comparisons done up to that time (including the time point under consideration). P values <0.05 were considered significant.

Post-hoc power analysis performed on the data collected by HPLC revealed that the sample size necessary to detect significant changes in unencapsulated bupivacaine concentrations over time ranged from 3 vials to >50 vials, depending on the pathogen and time point. Therefore, the information collected via HPLC is presented as raw or averaged raw values. Analyses were conducted using R version 4.1.3 with the lme4, lmerTest, and ggplot2 packages [13–16]. For these analyses, no outliers were identified or removed.

## Results

### Part one: Comparative microbial growth dynamics

**Fig 1:**
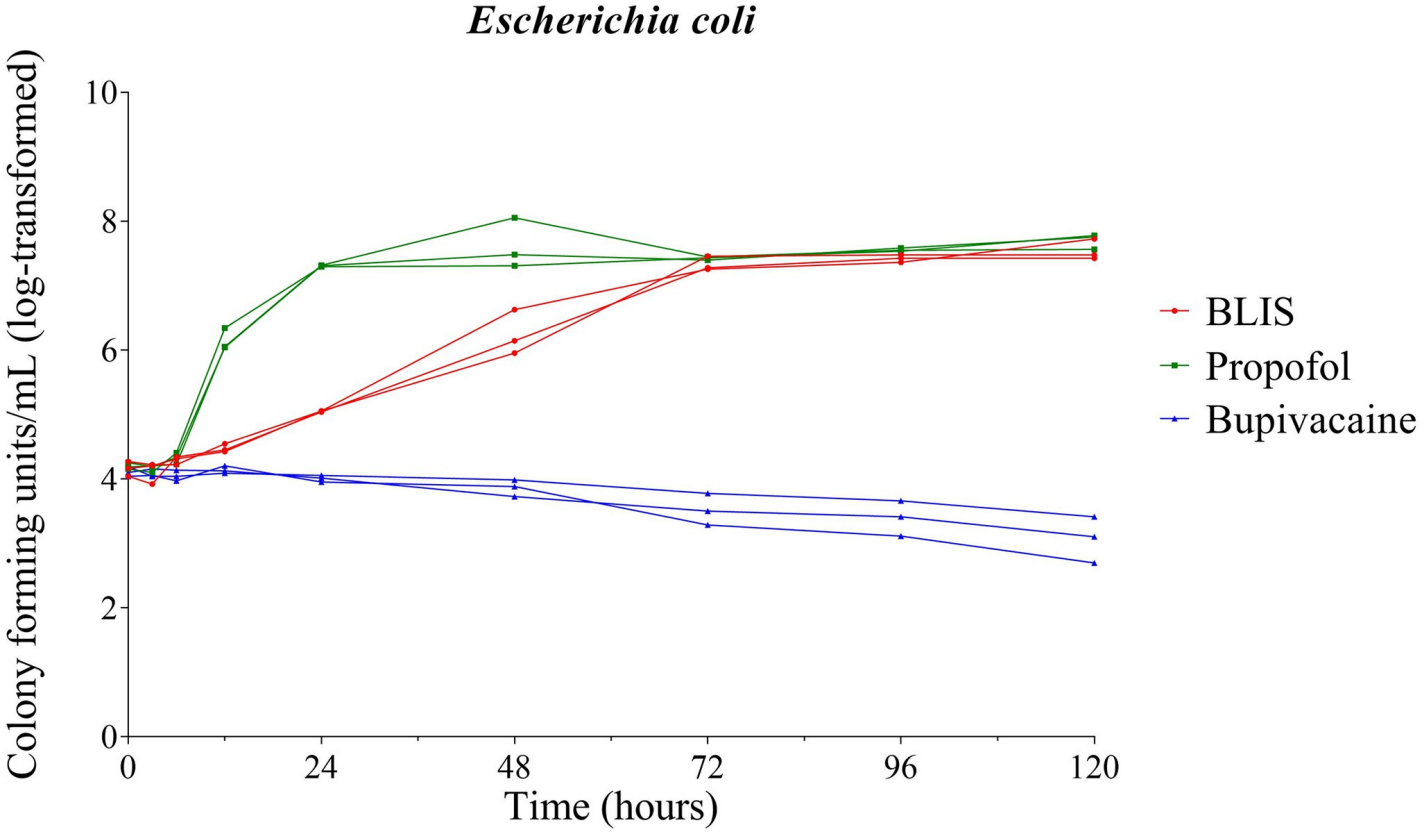
Growth dynamics of *Escherichia coli*. Growth of inoculated *Escherichia coli* over time in bupivacaine liposomal injectable suspension (BLIS), propofol, and bupivacaine 0.5%. Each line represents an individual vial; red lines represent BLIS, green lines represent propofol, and blue lines represent 0.5% bupivacaine.

Significant growth of *E*. *coli* in BLIS was observed starting 24 hours post inoculation (P = 0.019). Propofol also supported significant growth (P<0.0001) of *E*. *coli* 24 hours post inoculation and thereafter. No significant growth of *E*. *coli* occurred in bupivacaine 0.5% at any time point (Fig 1).

A significant difference between the growth of *E*. *coli* supported by BLIS and propofol was observed at the 24 (P<0.001) and 48 (P = 0.004) hour time points, with propofol supporting significantly more growth. At the 72 hour time point and beyond, no significant difference existed between growth dynamics of *E*. *coli* in BLIS and propofol (Fig 1).

**Fig 2:**
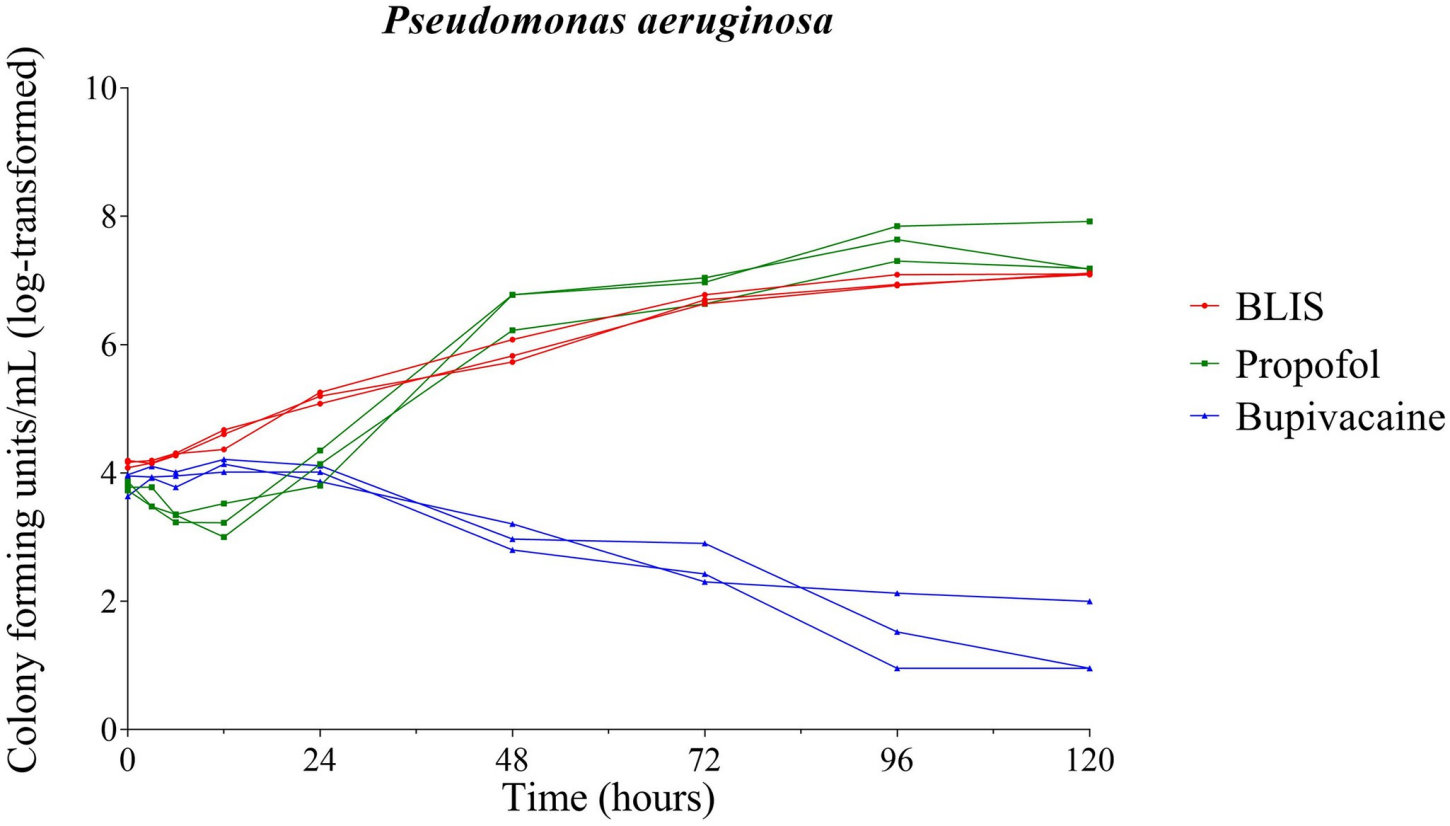
Growth dynamics of *Pseudomonas aeruginosa*. Growth of inoculated *Pseudomonas aeruginosa* over time in bupivacaine liposomal injectable suspension (BLIS), propofol, and bupivacaine 0.5%. Each line represents an individual vial; red lines represent BLIS, green lines represent propofol, and blue lines represent 0.5% bupivacaine.

Significant growth of *P*. *aeruginosa* in BLIS was observed beginning at 24 hours (P = 0.022). Propofol also supported significant growth of *P*. *aeruginosa* (P = 0.008) beginning 48 hours post inoculation and thereafter. No significant growth of *P*. *aeruginosa* occurred in bupivacaine 0.5% at any time point (Fig 2).

A significant difference between growth of *P*. *aeruginosa* supported by BLIS and propofol occurred at the 6 (P<0.001), 12 (P = 0.003), 24 (0.020), and 48 (P = 0.018) hour time points. At the 72 hour time point and beyond, no significant difference existed between growth dynamics of *P*. *aeruginosa* in BLIS and propofol (Fig 2).

**Fig 3:**
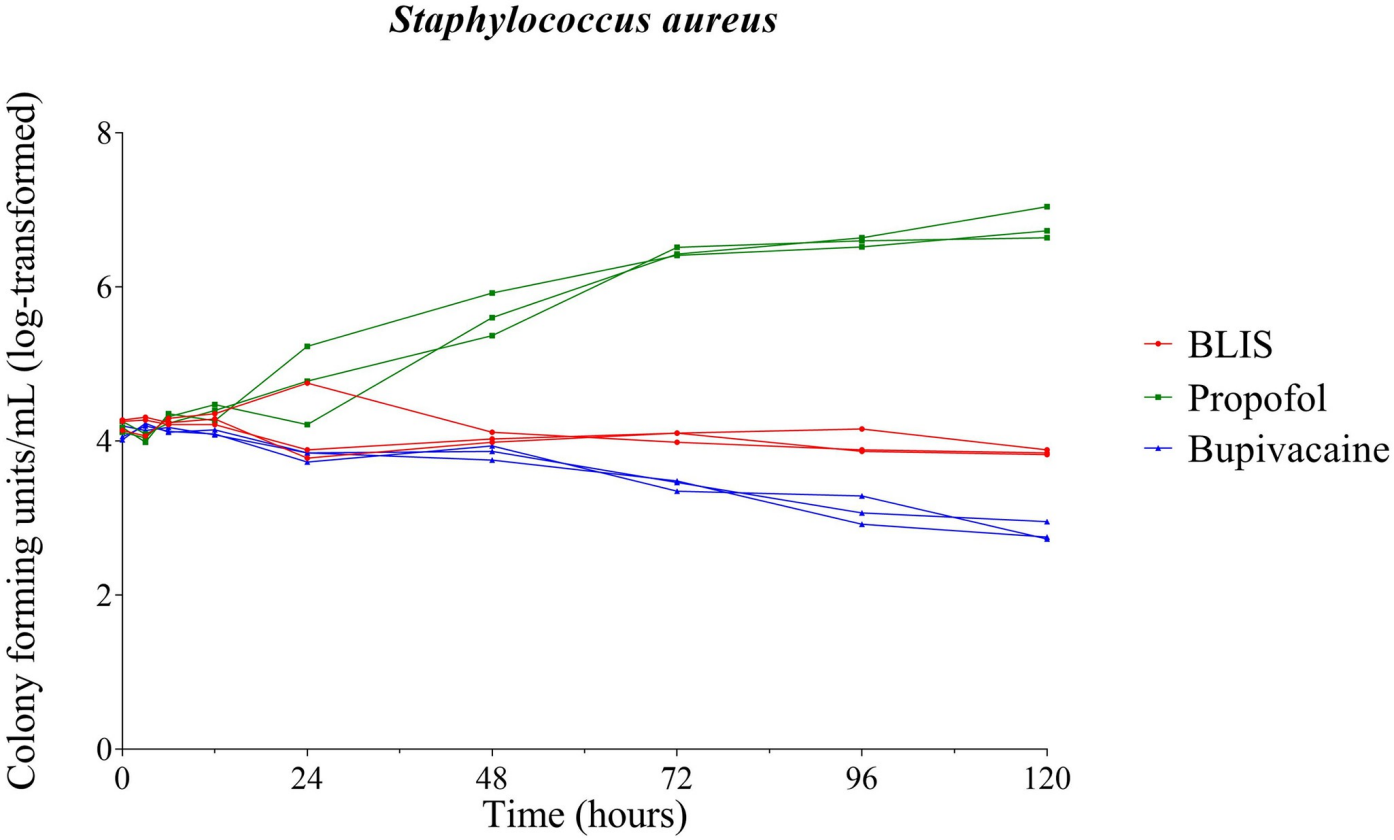
Growth dynamics of *Staphylococcus aureus*. Growth of inoculated *Staphylococcus aureus* over time in bupivacaine liposomal injectable suspension (BLIS), propofol, and bupivacaine 0.5%. Each line represents an individual vial; red lines represent BLIS, green lines represent propofol, and blue lines represent 0.5% bupivacaine.

No significant growth of *S*. *aureus* occurred in BLIS at any time point during this study. A significant reduction of *S*. *aureus* occurred in BLIS at 72 hours post inoculation (P = 0.038), and again at 120 hours (P = 0.046). Growth of *S*. *aureus* in propofol was significant (P = 0.0006) beginning 72 hours post inoculation. No significant growth of *S*. *aureus* occurred in bupivacaine 0.5% at any time point (Fig 3).

A significant (P<0.001) difference in concentration of *S*. *aureus* recovered from BLIS compared to bupivacaine 0.5% occurred at the 72 hour time point and beyond; the reduction in *S*. *aureus* was greater for 0.5% bupivacaine than for BLIS (Fig 3).

**Fig 4:**
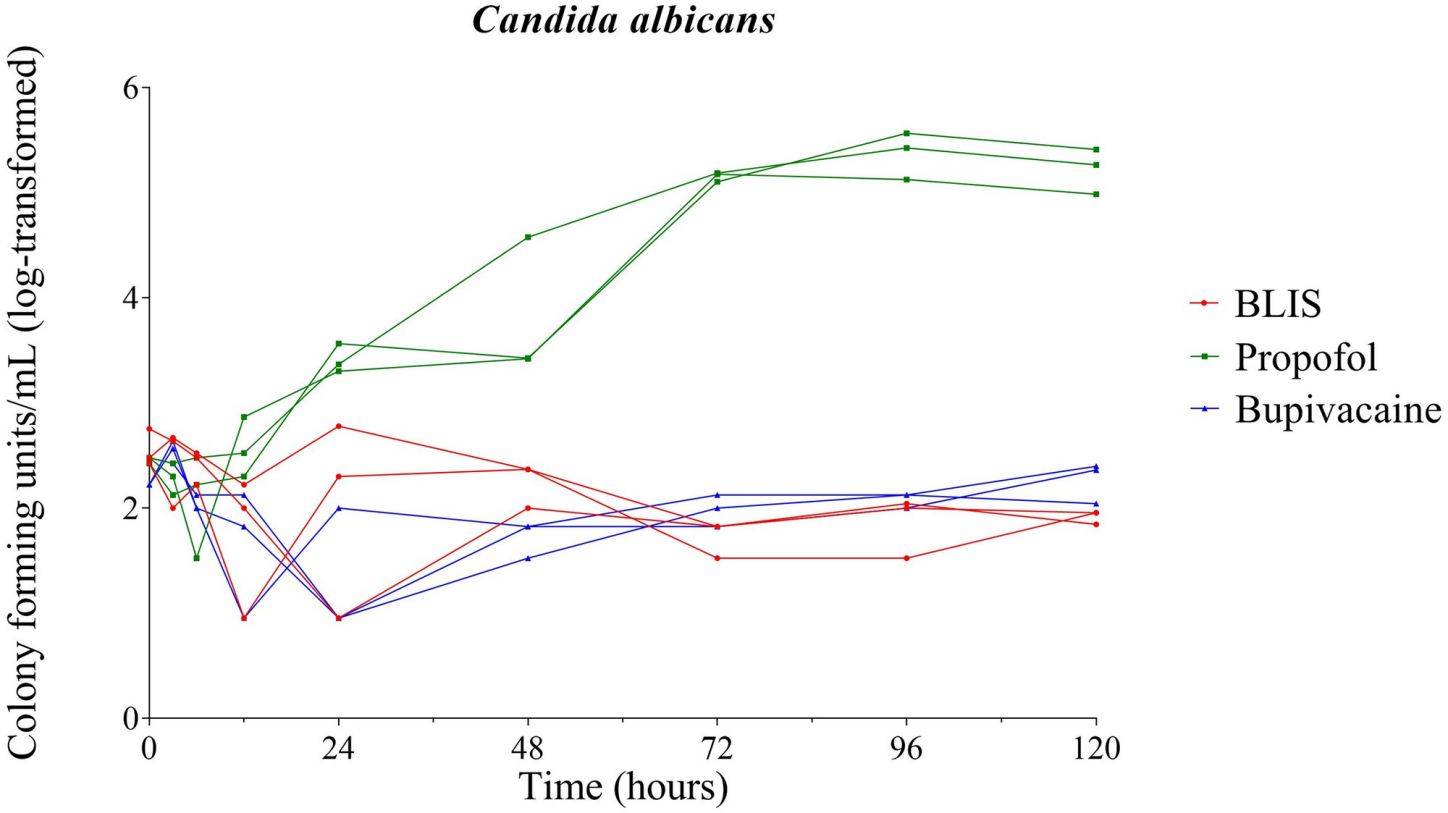
Growth dynamics of *Candida albicans*. Growth of inoculated *Candida albicans* over time in bupivacaine liposomal injectable suspension (BLIS), propofol, and bupivacaine 0.5%. Each line represents an individual vial; red lines represent BLIS, green lines represent propofol, and blue lines represent 0.5% bupivacaine.

No significant growth of *C*. *albicans* occurred in BLIS at any time point during this study. Propofol supported significant growth of *C*. *albicans* at 24 hours (P = 0.039) post inoculation, and significant growth was also observed at the 72 (P = 0.0007), 96, and 120 (P = 0.009) hour time points. No significant growth of *C*. *albicans* occurred in bupivacaine 0.5% at any time point (Fig 4).

A significant (P = 0.033) reduction in the concentration of *C*. *albicans* was observed at 120 hours post inoculation in BLIS compared to bupivacaine 0.5% (Fig 4).

### Part two: Unencapsulated bupivacaine concentrations

Changes in unencapsulated bupivacaine concentrations from baseline are reported in Table 1 as averaged, raw values ± one standard deviation.

**Table 1:** Average change from baseline in concentration of unencapsulated bupivacaine in μg/mL where treatment group data are the mean concentrations (n = 3) of three vials ± one standard deviation.

Time (hours)	*Escherichia coli*	*Pseudomonas aeruginosa*	*Staphylococcus aureus*	*Candida albicans*
0	934±20	854±128	845±144	672±52
24	925±36	1016±38	989±26	966±189
48	949±133	1290±377	939±26	1051±151
72	1018±104	880±22	947±48	852±123
96	1345±118	981±112	992±123	946±143
120	1555±356	1650±691	1021±43	1178±582

Time 0 represents the baseline unencapsulated bupivacaine concentration in bupivacaine liposomal injectable suspension prior to the introduction of microbial contaminants

## Discussion

The long acting local anesthetic BLIS is an attractive option for pain management due to its duration of action and lack of systemic side effects. However, economic pressures may result in the extra-label use of BLIS with multiple vial punctures over several days, potentially increasing the risk of microbial contamination.

The investigation of *in vitro* bacterial and fungal growth in BLIS is limited [17]. In the present study, trends in microbial growth supported by BLIS were pathogen specific such that BLIS supported the growth of *E*. *coli* and *P*. *aeruginosa* yet halted the growth of *S*. *aureus* and *C*. *albicans*. These findings are in disagreement with our hypothesis that BLIS would be bacterio- and fungistatic but are similar to those of a recent study that demonstrated that BLIS did not display the same bacteriostatic properties as traditional local anesthetics. Callahan et al. evaluated the *in vitro* growth of methicillin-resistant *Staphylococcus aureus*, methicillin-susceptible *Staphylococcus aureus*, coagulase-negative *Staphylococcus*, *E*. *coli*, and*Enterococcus* species in saline, bupivacaine 0.25%, lidocaine 1.0%, and BLIS. It was demonstrated that lidocaine and bupivacaine largely suppressed microbial growth when compared to saline (with the exception of methicillin-resistant *Staphylococcus aureus* species) while BLIS supported microbial growth similarly to saline and to a greater extent than local anesthetics (with the exception of *E*. *coli* in which BLIS supported growth to a much greater extent than saline) [17]. This differential support of growth may be due to the formulation of BLIS or the development of increased free bupivacaine concentrations following microbial contamination. BLIS contains 13.3 mg/mL of bupivacaine as well as cholesterol, 4.7 mg/mL, 1,2-dipalmitoyl-sn-glycero-3 phospho-rac-(1-glycerol) (DPPG), 0.9 mg/mL, tricaprylin, 2.0 mg/mL, and 1,2 dierucocylphosphatidylcholine (DEPC), 8.2 mg/mL. An identical product labeled for use in humans, Exparel, has a pH between 5.8 and 7.4 (Pacira Pharmaceuticals, San Diego, CA) [9, 18]. Carlson et. al. demonstrated that the concentration of unencapsulated bupivacaine in BLIS vials stored at room temperature for multiple days was between 1150 and 1200 μg/mL [10].

Propofol is a preservative-free, intravenous anesthetic induction agent containing soybean oil, glycerol, egg lecithin, and oleic acid with a pH between 6.0 and 9.0. The emulsion is labeled to be aseptically drawn into individual syringes with unused product to be discarded within six hours due to the potential for microbial growth and resultant infection (PropoFlo, Zoetis, Kalamazoo, MI) [19]. Robust *in vitro* microbial growth in propofol has been demonstrated [20–23], and propofol administration following inadvertent contamination and subsequent systemic inflammatory response syndrome has been reported in humans [24, 25]. The cause of vigorous bacterial growth in propofol and the association of propofol with nosocomial infection has not been explicitly described, but the lipophilicity of the ingredients has been suggested [24]. The current study illustrates significant growth of *E*. *coli*, *P*. *aeruginosa*, *S*. *aureus*, and *C*. *albicans* in propofol stored at room temperature and reinforces the importance of aseptic technique during handling and administration.

Bupivacaine is an injectable local anesthetic available in various concentrations that may contain preservatives or epinephrine. Some formulations are labeled for use from multidose vials stored at room temperature. Marcaine (Hospira, Lake Forest, IL), as utilized in the present study, does not contain an antimicrobial preservative nor epinephrine and has a pH between 4.0–6.5 adjusted with hydrochloric acid [26]. In previous studies, bupivacaine and numerous other local anesthetics have demonstrated bacteriostatic properties [12, 27–30]. The effects of drug concentration, temperature, preservatives, and duration of microbial exposure to anesthetic solution have been investigated [28]. Proposed mechanisms for the bacteriostatic action of local anesthetics include alterations in bacterial protein synthesis and changes in membrane permeability and subsequent cellular lysis [30]. Bupivacaine 0.5% did not support significant growth of any of the selected pathogens throughout the duration of the present study, consistent with previous investigations documenting the bacteriostatic properties of local anesthetics.

Bacterial and fungal growth and decay are dependent on numerous environmental factors including pH, temperature, water, oxygen, nitrogen, and carbon content [31]. *In vitro* lipid metabolism by microbial contaminants in propofol and BLIS may have affected growth dynamics [32], and the dissimilar lipid compositions of each drug may explain the differences seen in the rates of growth in our study. Additionally, the concentration of unencapsulated bupivacaine in BLIS may have halted growth of the fungal and gram-positive microbes to some extent when compared to propofol, given the previously demonstrated bacteriostatic properties of some local anesthetic formulations.

Statistical analyses were not performed on the HPLC data collected due to the small sample size, as this was only a secondary objective. The raw values generated by HPLC analysis reveal a small range in unencapsulated bupivacaine concentration between vials, even at baseline, with a mean increase in free bupivacaine concentrations over time. Interestingly, baseline values for unencapsulated bupivacaine were on average, lower than that reported by Carlson et al. [10], which may be attributable to batch-to-batch variability, as the HPLC samples were handled and analyzed similarly.

The small number of pathogens investigated and overall pathogen diversity is a limitation of this study as well as the inability to perform data analysis on samples collected for HPLC. Additional limitations exist in that microbial growth was evaluated at limited temperatures, and a saline control was not utilized. There likely exists a disparity between the inoculum size utilized in this study (0.5 McFarland standard) and the number of bacterial or fungal organisms that would be introduced into a vial of BLIS during clinically relevant contamination. Multiple factors contribute to the incidence of surgical site infection (as opposed to surgical site contamination), including perioperative hyperglycemia and urinary catheterization [33], therefore the findings of this study should not be extrapolated beyond the observation that BLIS variably supports microbial growth when extrinsically contaminated. Extra-label use of any drug that supports contaminant growth requires strong consideration as the prescriber may assume responsibility for negative outcomes.

## Conclusion

Bacterial and fungal growth dynamics in bupivacaine liposomal injectable suspension vary across time and organism, and BLIS supports significant growth of some gram-negative bacterial species. Unencapsulated bupivacaine concentrations, on average, increase minimally after *in vitro* contamination occurs. The clinical implications of these small changes in unencapsulated bupivacaine concentrations are unknown, but due to the demonstrated microbial growth over time, the use of BLIS beyond the approved Food and Drug Administration label should only be undertaken with caution and with adherence to strict aseptic technique. Extra-label use of BLIS has been suggested by previous studies and anecdotal experience; however, the potential for contamination and subsequent microbial growth should be strongly considered when multiple vial punctures occur over several days. To economically incorporate BLIS into multimodal pain management protocols, while also considering patient safety, it is our suggestion that practitioners weigh the risks versus benefits when handling BLIS in an extra-label manner.

## Supporting information

S1 File Average and log-transformed bacterial and fungal colony forming unit counts. Values for the number of colony forming units present in artificially inoculated bupivacaine liposomal injectable suspension, bupivacaine 0.5%, and propofol. The triplicate count of the highest dilution with a countable range (target 20 to 200 colony forming units) was averaged for each drug.
(XLSX)

S2 File Change from baseline concentration of colony forming units by organism represented as p values in three contaminated vials of bupivacaine liposomal injectable suspension (n = 3). (DOCX)

S3 File Raw value changes from baseline in concentration of unencapsulated bupivacaine in μg/mL in twelve vials of artificially inoculated bupivacaine liposomal injectable suspension. (XLSX)
