# Supplementary material for: Comparative growth dynamics of bacterial and fungal contaminants in bupivacaine liposomal injectable suspension, bupivacaine 0.5%, and propofol
Source: PLoS One. 2023 Feb 16;18(2):e0281768. doi: 10.1371/journal.pone.0281768 (PMC9934369; doi:10.1371/journal.pone.0281768)
Supplement: S2 File — (DOCX) [file pone.0281768.s002.docx]

**Change from baseline concentration of colony forming units by organism represented as p values in three contaminated vials of bupivacaine liposomal injectable suspension (n=3).**

| **Time (hours)** | ***Escherichia coli*** | ***Pseudomonas aeruginosa*** | ***Staphylococcus aureus*** | ***Candida***  ***albicans*** |
| --- | --- | --- | --- | --- |
| 3 | 1.0000 | 1.000 | 1.000 | 1.0000 |
| 6 | 1.000 | 0.2945 | 1.000 | 1.0000 |
| 12 | 0.0829 | 0.0926 | 1.000 | 1.0000 |
| 24 | 0.0196 ^a^ | 0.0224^a^ | 1.000 | 1.0000 |
| 48 | 0.0246 ^a^ | 0.0259^a^ | 0.9355 | 1.0000 |
| 72 | 0.0036^a^ | 0.0031^a^ | 0.0078^a^ | 0.0888 |
| 96 | 0.0029 ^a^ | 0.0038 ^a^ | 0.5204 | 0.2951 |
| 120 | 0.0006 ^a^ | 0.0006 ^a^ | 0.0744 | 0.1719 |

^a^ Represents a statistically significant change (p value < 0.05) from baseline inoculum value
